# Unusual primary osseous Hodgkin lymphoma in rib with associated soft tissue mass: a case report and review of literature

**DOI:** 10.1186/1746-1596-7-64

**Published:** 2012-06-08

**Authors:** Yang Li, Xiao-bing Wang, Xiao-ying Tian, Bin Li, Zhi Li

**Affiliations:** 1 Department of Pathology, The First Affiliated Hospital, Sun Yat-sen University, 58, Zhongshan Road II, Guangzhou, 510080, China; 2 Department of Pathology, Guangdong General Hospital, 106, Zhongshan Road II, Guangzhou, 510080, China; 3 School of Chinese Medicine, Hong Kong Baptist University, 7, Baptist University Road, Kowloon Tong, Hong Kong

**Keywords:** Hodgkin lymphoma, Primary bone tumor, Lymphoma, Differential diagnosis

## Abstract

Hodgkin lymphoma (HL) typically presents as nodal lesion and may involve extranodal sites during the progression of the disease. Primary osseous HL without any lymph node association is extremely rare and only a few such cases have been described in the literature. We present a case of unusual primary HL in rib occurring in a middle-aged female patient. Computed tomography (CT) scan revealed an osteolytic lesion was located at the right second rib and was associated with a large soft tissue mass. There was no regional lymph node involvement. CT scan of neck and abdomen was performed and showed no pathologic findings, particularly no lymphadenopathy and organomegaly could be observed. Histologically, typical binucleated Reed-Sternberg (RS) cells and lacunar cells were scattered in the background of reactive inflammation with infiltration of lymphocytes, histiocytes and eosinophilic granulocytes. By immunohistochemistry, RS cells and lacunar cells were positive for CD15 and CD30 with typical membrane and paranuclear dot-like staining pattern. However, these cells were negative for Epstein-Barr virus detection by *in situ* hybridization. A diagnosis of primary osseous HL was made. The patient received systemic chemotherapy and local radiotherapy, and was on regular follow-up for 24 months. There was no sign of recurrence of tumor and lymph node or bone marrow involvement. Because there is a possibility of secondary bone involvement by systemic HL, strict histological analysis and thorough radiographic examination are suggested to be necessary for accurately diagnosing this tumor when it presents as a solitary bone lesion.

**Virtual slides:**

The virtual slide(s) for this article can be found here:

http://www.diagnosticpathology.diagnomx.eu/vs/2846916171507084

## Background

Hodgkin lymphoma (HL) most commonly occurs in cervical lymph nodes, and the extranodal forms are rare accounting for less than 1% [1-3]. Typically, HL is a systemic disease in which 10% to 20% of cases show involvement of the bone during the progression of the disease [4-6]. However, primary osseous HL without any lymph node association is extremely rare and so far only a few such cases with immunohistochemical and/or molecular confirmation have been described in the literature, with none of these cases being from China [7-20]. According to Ann Arbor classification, primary extranodal HL is considered at stage I, but systemic HL with secondary bone involvement corresponds to stage IV of the disease. Therefore, it is indeed a challenge for clinicians to make a correct diagnosis when the HL presents as a solitary bone mass because inaccurate diagnosis may lead to delays in clinical treatment. Herein we describe an additional case of primary osseous HL arising in the rib in a middle-aged female patient without any lymphatic manifestation.

## Case presentation

### Clinical presentation and management

**Figure 1:**
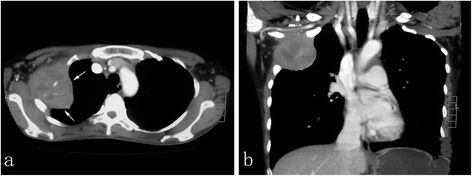
**Radiographic examination of the lesion.** (**a**) Osteolytic lesion of the right second rib with associated soft tissue mass appeared to have a distinct border to the lung in periphery in computed tomography scan (white arrow). (**b**) The osseous destruction of rib showed adjacent soft tissue extensions with moderate gadolinium enhancement but no involvement of regional lymph node was observed.

A 38-year-old Chinese female presented with pain at the right upper side of the chest and adjacent soft tissue swelling for 3 months. A week before admission to our hospital, she was suffering from a gradually severe pain at the chest and fever. As a result, the patient was referred to our hospital for examination and treatment. Physical examination showed the patient had a mild soft tissues edema at her right upper lateral chest wall and severe pain was elicited upon chest pressure. The patient had a low grade fever of 37.4°C. There was no weight loss and no palpable lymphadenopathy or organomegaly. The laboratory results, including blood count, differential, liver and renal function, were within the normal range. A computed tomography (CT) scan of the chest revealed a single osteolytic lesion in the right second rib with associated soft tissue mass, measuring 6.0 × 5.0 × 5.0 cm in size. The most of rib was observed to be destroyed and the involvement of adjacent pleura and right upper lobe of lung were also noted (Figure 1). The lesion showed moderate enhancement after gadolinium injection. There was no enlarged lymph node found in mediastinum and thoracic cavity. A CT scan of neck and abdomen showed no pathologic findings, particularly no lymphadenopathy could be observed. A CT guided needle biopsy was performed initially, but histopathological examination showed pieces of fibrosis with infiltration of inflammatory cells. Therefore, a surgical biopsy followed by a microscopic examination was performed. After diagnosis, the patient underwent polychemotherapy according to a modified COPP protocol (bleomycin, etoposide, adriamycin, cyclophosphamide, vincristine, oncovin, procarbazine, prednisone) for five cycles before initiation of involved field radiotherapy. The lesion was regressed, and the patient was on regular follow-up for 24 months after radiotherapy. The bone marrow examination was performed at 6 months after radiotherapy, there was no abnormality found. During the period of following-up, there was no sign of recurrence of tumor and lymph node enlargement.

## Material and methods

The surgical biopsy was routinely fixed in 10% neutral buffered formalin. The tissues were embedded in paraffin. Four micrometer-thick sections were stained with H&E. Immunohistochemical analyses were performed using the ChemMate Envision/HRP Kit (Dako, Glostrup, Denmark). The antibodies used in this study included a broad panel of antibodies against CD1a, CD3, CD4, CD8, CD15, CD20, CD30, CD38, CD43, CD45,CD56, CD79a, ALK, Pax-5, BOB-1, OCT-2, S-100 protein, pan-cytokeratin and epithelial membrane antigen (EMA). Epstein-Barr virus (EBV) infection was also assessed by *in situ* hybridization for EBERs according to the manufacturer’s instruction (Dako, Glostrup, Denmark). The antibodies were obtained from Dako Cytomation, Santa Cruz Biotechnology (Santa Cruz, CA, USA) and Novocastra laboratories LTD (Hong Kong, China).

## Results and discussion

**Figure 2:**
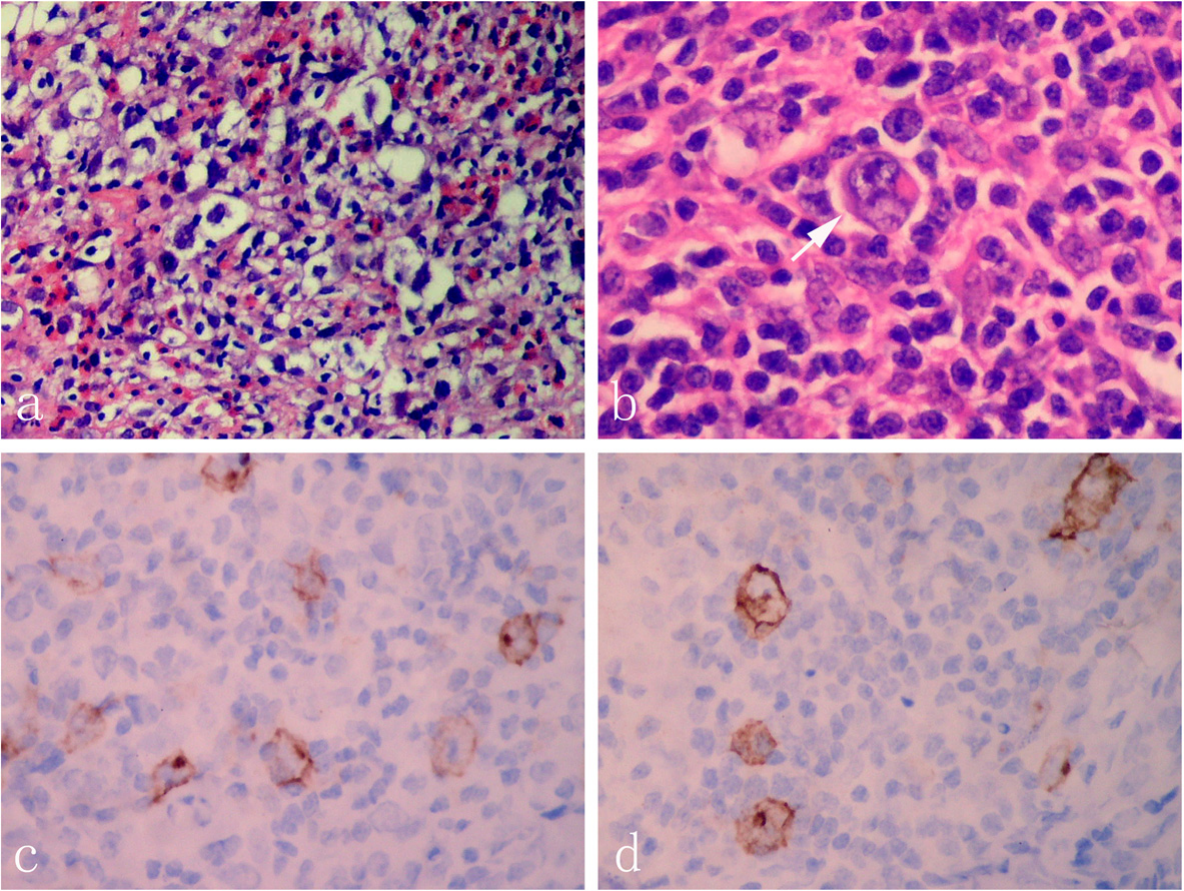
**Photomicrographs of the osseous lesion.** (**a**) Scattered lacunar cells were observed in the inflammatory background with numerous lymphocytes, histiocytes, and eosinophilic granulocytes. (**b**) Typical morphology of Reed-Sternberg cells, with large cytoplasms and prominent eosinophilic nucleoli were also found in the lesion (white arrow). The immunohistochemical staining of the RS cells confirmed the expression of CD30 (**c**) and CD15 (**d**) with typical membrane and paranuclear dot-like staining pattern (a-b, HE staining with original magnification × 400; c-d, immunohistochemical staining with original magnification × 400).

Under microscopic examination, the lesions were characterized by a fibrosis developing in bone marrow spaces, which was infiltrated with diffuse lymphocytes, plasma cells, histiocytes, and eosinophilic and neutrophilic granulocytes. Typical morphology of Reed-Sternberg (RS) cells, with large cytoplasms and prominent eosinophilic nucleoli, were scattered in the background of reactive inflammation. Lacunar cells, mummified cells and small foci of acidophilic necrosis were also found in the tissue. The immunohistochemistry staining of the RS population confirmed the expression of PAX-5, CD30 and CD15. However, these cells did not express CD1a, CD3, CD4, CD8, CD20, CD38, CD43, CD45, CD56, CD79a, ALK, BOB-1, OCT-2, S-100 protein, pan-cytokeratin and EMA (Figure 2). Epstein-Barr virus (EBV) status was negative. Taken together, these morphological and immunological data were consistent with a classical Hodgkin’s lymphoma, mixed cellularity, according to the WHO diagnostic criteria [1].

Primary classical Hodgkin lymphomas (HLs) are well established lymphoproliferative neoplasms of the lymph nodes with bimodal age curve. The extranodal HLs are rare and account for less than 1%. The most common site of extranodal HLs is gastrointestinal tract, followed by the pulmonary system, thyroid, skin, genitourinary system, and central nervous system. Although osseous involvement could be observed in the late stages of HLs, it is extremely rare for patients to present with primary HLs of the bone. To our knowledge, so far primary osseous HL has been reported in approximately 30 patients in the literature. Some of the cases have been associated with extra-osseous involvement. Primary osseous HL confined to the bone is so rare that approximately 19 cases have been reported worldwide to date, only 12 patients with primary solitary osseous HL [7-14] and 7 patients with primary multifocal osseous HL disease have been reported without lymphatic manifestations [15-20]. Moreover, primary osseous HL presenting as a bone lesion with an associated soft tissue mass is quite unusual [10,21-23]. Histologically, mixed cellularity and nodular sclerosis variant of classical HL are most common subtype of primary osseous HL in literature. In clinical, four types of osseous involvement by HL may be presented: (1) primary osseous HL with solitary lesion; (2) primary osseous HL with multifocal lesion; (3) HL simultaneously in osseous and non-osseous sites; and (4) recurrence of HL at osseous sites.

Since cases of primary osseous HL are so rare, the diagnosis should be only made by strict histological and clinical manifestation. According to the WHO criteria, a diagnosis of primary bone lymphoma must be (1) a single skeletal tumor, with or without regional lymph node involvement, and (2) multiple bone lesions without visceral or lymph node involvement [24]. Many previously described cases of primary extranodal HL appeared to have concurrent and/or subsequent nodal involvement. They are factually secondary cases in a context of systemic HL. The word "primary" should be used cautiously. In the present case, we found: (1) no superficial lymphadenopathy presented at the time of diagnosis; (2) chest, neck and abdomen radiological studies showed no involvement of lymph nodes in mediastinum, thoracic and abdominal cavity; (3) the complete blood count and differential are within the normal limits; (4) the lesion was predominant in bone with associated soft tissue mass, without positive adjacent lymph nodes; (5) live, spleen and bone marrow were free of disease at the time of diagnosis and the period of following-up; (6) the RS cells in tumor demonstrated a typical immunohistochemical profile with CD30, CD15 and PAX-5 positive. CD45, CD20 and other T-cell lineage markers were negative. These results prove the present case to be of primary osseous origin. The recent studies suggest that 2-[18 F]-fluoro-2-deoxy-d-glucose positron emission tomography/computed tomography (18 F-FDG-PET/CT) enables systemic HL with secondary bone invasion to be distinguished from primitive osseous HL. This technique is highly specific in demonstrating the isolated osseous localization of the tumor and should be recommended in all patients with putative osseous lymphoma [25].

Regarding the cellular origin of RS cells in HLs, especially for those in extranodal lesions, it remains to be determined whether RS cells are clonal B cells that have lost their B cell phenotype due to destructive somatic mutation [26]. Although recent investigations have defined a characteristic profile of recurrent copy number gains and losses in classical HL, including gains of chromosomes 2p, 9p, 16p, and 17q and losses of 13q, 6q, and 11q, cytogenetic and molecular studies of HL are limited. Several recurrent genetic lesions have been identified in RS cells, and most of them affect members of the JAK/STAT or NF-κB signaling pathways [27]. It is likely that EBV plays a role in reprogramming and survival through dysregulation of several signaling networks and transcription factors, including inactivating mutations in the TNFAIP3 tumor suppressor gene and encoding a negative regulator of NF-κB activity [28]. The RS cell furthers its own survival by attracting a supportive microenvironment of immune and stromal cells, and suppressing local immune responsiveness. Although many questions remain unanswered, a better understanding of cellular interaction may help to clarify the pathogenesis of HL and promote novel approaches for targeting the therapy of this malignant tumor.

Osseous HLs usually show osteolysis in radiological examination, it is often misdiagnosed as osteomyelitis [10,16,29]. The present case exhibited either osteolytic lesion with soft tissue mass or inflammatory background with eosinophilic granulocytes infiltration, which might sometimes cause diagnostic confusion with primary bone sarcoma, eosinophilic granuloma and non-Hodgkin lymphoma of bone. However, osteomyelitis and primary bone sarcoma lack the specific RS cell with immunoreactivity for CD30 and CD15. Eosinophilic granuloma of bone shows clonal neoplastic proliferation of Langerhans cells that express CD1a, S-100 and Langerin. The incidence of non-Hodgkin lymphoma of bone is higher than that of primary osseous HL. The most common subtype of non-Hodgkin lymphoma in bone is diffuse large B-cell lymphoma. Immunohistochemically, most of non-Hodgkin lymphomas of bone are positive for CD45 and B-cell or T-cell lineage markers with or without CD30 expression. Co-expression of CD15 and CD30, but lacking CD45 expression of tumor cells may be helpful to confirm the diagnosis of osseous HL. Although the detection of EBV-encoded RNA (EBER) or LMP1 is indicative of classical HL, EBV is found in only a proportion of cases, particularly in mixed cellularity and lymphocyte depleted classical HL with the highest frequency of 75% [1]. By contraries, some subtypes of non-Hodgkin lymphoma, including extranodal NK/T cell lymphoma and EBV positive diffuse large B-cell lymphoma, show EBV infection in tumor cells. The present case showed EBV negative by *in situ* hybridization although it presented typical histological and immunohistochemical profile of classical HL. Therefore, detection of EBV-encoded products in tumor is not a definitive point to confirm the diagnosis of HL. In addition, classical HL has to be distinguished from anaplastic large cell lymphoma (ALCL) in such an unusual extranodal location because both tumors show CD30 immunoreactivity with typical membrane and paranuclear dot-like staining pattern [30]. However, differential diagnosis between HL and ALCL can be made by expression of anaplastic lymphoma kinase (ALK), EMA and CD45 which are typical of ALCL, ALK-positive, while co-expression of CD30 and PAX5 is very helpful in differentiating HL from ALCL, ALK-negative.

## Conclusion

In conclusion, only a few cases of primary osseous HL have been reported in the literature. Our additive case is also presented for its rarity of site. It is the first case of primary osseous HL occurring in Chinese. The diagnosis of primary osseous HL is difficult and should be made cautiously. Besides confirmation by strict histopathological and immunohistochemical analysis, radiographic examination is helpful for demonstrating potential extra-osseous lesion to distinguish systemic HL with secondary bone invasion from primary osseous HL. In clinical, an immediate diagnosis at the early stage of disease and timely treatment with systemic chemotherapy and local radiotherapy are required because the patients with primary osseous HL appear to have a good long-term prognosis when the lesion is restricted in bone without systemic dissemination.

## Consent

Written informed consent was obtained from the patient for publication of this case report and any accompanying images. A copy of the written consent is available for review by the Editor-in-Chief of this journal.

## Competing interests

The authors declare that we have no competing interests.

## Authors' contributions

YL and XB W made contributions to acquisition of clinical data, and analysis of the histological features by H&E staining they are joint first co-authors and made an equal contribution to this work. XYT drafted the manuscript. ZL revised manuscript critically for important intellectual content and had given final approval of the version to be published. BL carried out the immunoassays. All authors read and approved the final manuscript.
